# High Performance Integration Pipeline for Viral and Epitope Sequences

**DOI:** 10.3390/biotech11010007

**Published:** 2022-03-21

**Authors:** Tommaso Alfonsi, Pietro Pinoli, Arif Canakoglu

**Affiliations:** 1 Dipartimento di Elettronica, Informazione e Bioingegneria, Politecnico di Milano, Via Ponzio 34/5, 20133 Milano, Italy; pietro.pinoli@polimi.it (P.P.); arif.canakoglu@policlinico.mi.it (A.C.); 2 Policlinico di Milano Ospedale Maggiore, Fondazione IRCCS Ca’ Granda, Via Francesco Sforza, 35, 20122 Milano, Italy

**Keywords:** data integration, viral datasets, sequence analysis, SARS-CoV-2, COVID-19, bioinformatics, metadata management

## Abstract

With the spread of COVID-19, sequencing laboratories started to share hundreds of sequences daily. However, the lack of a commonly agreed standard across deposition databases hindered the exploration and study of all the viral sequences collected worldwide in a practical and homogeneous way. During the first months of the pandemic, we developed an automatic procedure to collect, transform, and integrate viral sequences of SARS-CoV-2, MERS, SARS-CoV, Ebola, and Dengue from four major database institutions (NCBI, COG-UK, GISAID, and NMDC). This data pipeline allowed the creation of the data exploration interfaces VirusViz and EpiSurf, as well as ViruSurf, one of the largest databases of integrated viral sequences. Almost two years after the first release of the repository, the original pipeline underwent a thorough refinement process and became more efficient, scalable, and general (currently, it also includes epitopes from the IEDB). Thanks to these improvements, we constantly update and expand our integrated repository, encompassing about 9.1 million SARS-CoV-2 sequences at present (March 2022). This pipeline made it possible to design and develop fundamental resources for any researcher interested in understanding the biological mechanisms behind the viral infection. In addition, it plays a crucial role in many analytic and visualization tools, such as ViruSurf, EpiSurf, VirusViz, and VirusLab.

## 1. Introduction

Following the outbreak of the SARS-CoV-2 pandemic, many scientists are involved in the study of the genetic mechanism at the foundation of viral spread and infection. The ability to catch useful insights from the observation of data is crucial and represents a difficult challenge, especially in the context of the pandemic due to the rapid evolution of the viral genome. With thousands of SARS-CoV-2 viral sequences being captured every day across the globe, and without a commonly agreed standard for the encoding of information, it is certainly hard to make sense of such an outstanding quantity of data. In order to access the information in a uniform and valuable way, powerful and scalable methods for management and integration of viral data are needed. Addressing this issue, in 2020 we presented ViruSurf [1], an integrative search system for viral sequences that contain samples of SARS-CoV-2, as well as SARS-CoV, MERS-CoV, Ebola, and Dengue viruses, collected from several data sources. We designed ViruSurf to be as extensible as possible. It now includes information about several viral species, described through their viral sequences, individual sequence changes, both at the nucleotide and amino acid level, functional annotations, and curated metadata information about their hosts, the sequencing process, and the experiment. More recently, the system has been extended with EpiSurf [2] to also include epitope region annotations, and with VirusViz and VirusLab, two visualization interfaces. While in our previous works [1,2,3] we primarily discussed the features of the systems ViruSurf, EpiSurf, and VisusLab from the user’s perspective, in this manuscript we intend to address the technological aspects behind their realization. Moreover, after almost two years since the launch of ViruSurf and EpiSurf and millions of sequences integrated, we can now prove the scalability of the methods adopted, especially in light of the 64× increase in the volume of SARS-CoV-2 sequences registered during the year 2021.

## 2. Related Works

Several integrative search systems for viral sequence exist, even if we do not have enough documentation on their internals to fully understand how they compare to our work. Among the most notable, we found 2019nCoVR [4] at the Chinese National Genomics Data Center, CoV-Seq [5], and CARD [6]. They rely on the sequences imported from the most important database institutions, with GISAID [7], NCBI [8], and CNGB [9] as the principal data sources. Their underlying pipelines perform metadata integration, duplicate sequence management, and variant calling; 2019nCoV and CoV-Seq also include the quality assessment of the sequences, genome annotation, and variant effect prediction; finally, only 2019nCoV includes viral samples belonging to coronaviruses other than SARS-CoV-2. Compared to these, ViruSurf and EpiSurf offer more powerful query and search capabilities. Indeed, our systems are supported by a conceptual model that describes and links many aspects of the isolates imported by our data pipeline; such a general approach also reflects the possibility to integrate different types of viruses. We already provide samples of multiple families of viruses (SARS-CoV-2, SARS-CoV, MERS-CoV, Dengue, and Ebola Virus) and query heterogeneous types of information, i.e., epitopes in addition to viral sequences.

## 3. Materials and Methods

### 3.1. Data Sources

The sources from which we download and integrate viral sequences, along with related information, into ViruSurf are COG-UK [10], NMDC, and GISAID for the isolates, and NCBI (which includes the sources GenBank and RefSeq) ([11,12]) for both isolates and taxonomy. While in [1] we already discussed such data sources in detail, here we focus on the most technical aspects related to the download of updated sequences and annotations.

From NCBI, we regularly download the list of sequences, identified by the accession_id, and compare it with the sequence identifiers available in our database. Once the identifiers have been obtained, we then download the novel viral sequences from the Nucleotide database and the hosts’ metadata from the Biosample database through the Entrez API. The integration of these samples inside our database starts as soon as the first sample is downloaded and proceeds in parallel with the download process.

Additionally, we periodically download updates for the sequences of COG-UK as a pair of files: a Multi-FASTA file for nucleotide sequences and a CSV for the samples’ metadata. Between those files, the data are linked through the accession_id of the sequences.

For updating NMDC, we download the nucleotide sequence of each sampled sequence in FASTA format from the repository at http://nmdc.cn/SProject/virus/genbankfasta/ (accessed on 13 October 21). Knowing the accession_id, we then collect the associated metadata by downloading the JSON file from the NMDC website, in particular the web page describing the sequence.

Finally, we collect the latest updates from GISAID by downloading a JSON export of the source’s database. The file includes metadata and amino acid changes for each sequence, but not the nucleotide sequences itself. Indeed, the access to GISAID’s private repository is subject to limitations; as such, the availability and continuous integration of GISAID’s sequences may change over time and we cannot guarantee regular updates. In addition, GISAID does not allow to transform its data and integrate it with other sources. For these reasons, we limit the processing of GISAID data to the mapping of the original information to our schema, exploiting the power of the conceptual model.

Besides these data sources, we also retrieve additional structural annotations (portions of viral proteins), called epitopes, from the Immune Epitope Database (IEDB) [13]. The amino acid changes affecting the epitope regions can dramatically reduce the capacity of a host organism to recognize viral proteins and thus defend the organism from the infection. The IEDB collects these regions and shares them as public CSV files.

While viral sequences are the main subject of the integration pipeline, we are also interested in connecting such entities to the correct taxonomy category. The NCBI’s taxonomy database [14] is the principal resource used by our pipeline to retrieve the unique name and identifier of every organism (both viral and host). Furthermore, at the beginning of the integration process, the reference sequence of any new virus species is fetched first from the NCBI’s RefSeq database. The preferred method for accessing the NCBI resources is through the Entrez API [15], which provides a convenient way to query every NCBI database and download files as XMLs (or INSDSeq XMLs for isolates).

### 3.2. Data Model

**Figure 1:**
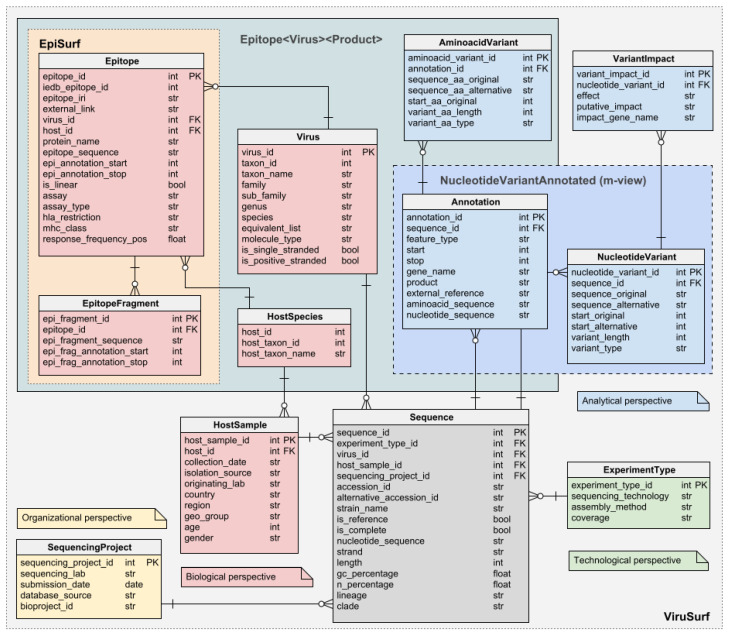
The Viral Conceptual Model (VCM): the central fact Sequence is described by the four different dimensions (biological, technical, organizational, and analytical). The first three are retrieved by the metadata, while the last one (analytical) is computed by processing the sequence through the pipeline. Part of the biological dimension is the two epitopes’ tables (Epitope and EpitopeFragment. The schema also anticipates two additional data structures, Epitope<Virus><Product> and NucleotideVariantAnnotated, which are the subjects of Section 3.5.3 and Section 3.5.4.

The Viral Conceptual Model, or VCM, is at the core of the (Web) services we implement to cope with SARS-CoV-2 sequences. VCM is a star schema-based conceptual model where the sequences (facts) are represented in terms of metadata and extrapolated data (dimensions or perspective). Figure 1 reports the structure of the model with the four dimensions highlighted. The data of the *Biological*, *Organizational*, and *Technological* dimension are extrapolated by parsing the metadata associated to each sequence and describe the species of the virus and the biological and demographic information of the host from which the sequence has been isolated, the submission information (including the date), and the sequencing related data, respectively. The entities Epitope and EpitopeFragment enrich the *Biological* dimension with the annotations extrapolated from IEDB; as they are specific to the combination of virus, protein, and host under consideration, they are linked to the Virus, Annotation, and HostSpecies entities. On the other hand, the Analytical dimension has been designed to represent those pieces of information that can only be retrieved by processing the sequence itself. Those include the annotated areas of the viral genome that are present in the sequence, the nucleotide mutations along with their putative impact, and the amino acidic mutations in the products of the genes in the sequences.

Notice that VCM (a) does not restrict to SARS-CoV-2, but it is extensible to virtually any viruses (as a matter of fact, some versions of the databases we build on VCM also store data of other viruses such as Dengue and Ebola) and (b) it is a mere conceptual model and it is not bound to any particular technology, indeed some of our applications store VCM data within relational databases while others store JSON documents.

### 3.3. Metadata Transformation

The main goal of the metadata pipeline is to map the metadata information available for each sequence to the corresponding attribute in the VCM. In most cases, in order to import the metadata of a single sequence, it is enough to download the corresponding record, read and parse it according to the file format, and, finally, store the attributes of interest as rows of the database, using the proper foreign keys to connect the entities. Besides the apparently simple process, the implementation is complicated by the countless differences between the data sources in terms of quality, quantity, and structure of the data. Thus, four different metadata pipelines have been implemented to embed the knowledge from each source into the process of finding, extracting, and transforming the attributes of interest for the data integration process. Compared to the others, the pipeline integrating NCBI sequences is undoubtedly the longest one, as the origin has slowly started to separate the information about the host from the sequence’s file, requiring to query, download, and transform an additional XML record from the NCBI Biosample database [16] for each host. Instead, other sources, such as GISAID or COG-UK, compress all their database into one or two files, and—as NMDC—they use simpler formats like JSON, CSV, Fasta, or Multi-FASTA that are easier to handle in Python (the language used for our pipeline); as such, their transformation is straightforward.

Several data quality aspects regarding metadata are harmonized during the sequence integration process. For example, the pipeline corrects misspellings in the names of organisms as well as USA counties. Then, the attributes host_taxon_name, gender, product (protein’s name) and all the dates are parsed and rewritten to reduce the presence of synonyms; differing date formats are homogenized. Moreover, in some cases, we observed incoherent or wrong assignments in the imported data between countries and continents, especially regarding intercontinental states. Therefore, we started to curate the value of geo_group using a dictionary that maps countries to continents without ambiguity and prevents misspells. Finally, the pipeline computes the missing metrics, i.e., the length of the sequence, the ratio of G and C pairs over the sequence of nucleotides (GC%), and the number of unknown bases (N%).

In this section we discuss most of the operations necessary to the transformation and integration of the metadata originating from the sources. A few more steps are necessary to compute the missing lineage annotations (relative to the sequences imported from NMDC and NCBI) and to recognize the duplicate sequences that have been imported from multiple sources. For performance reasons, these operations are carried on at the end of the sequence integration process. The discussion of these, and other mandatory steps for the finalization of the database, is postponed to Section 3.5.

**Figure 2:**
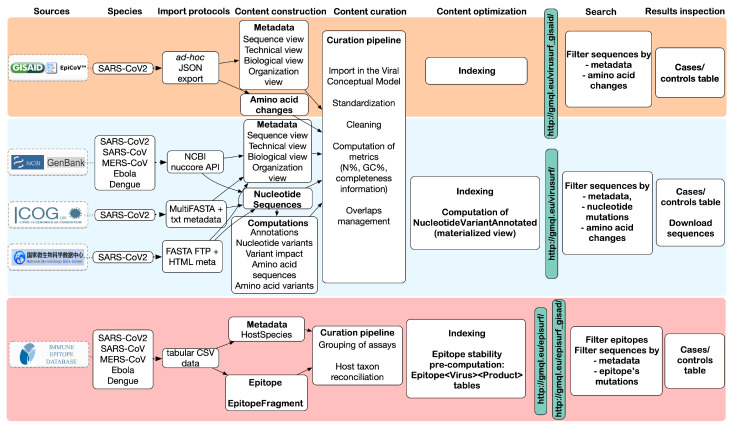
Overview of the data flow and pipeline stages for the integration of viral sequences into ViruSurf and EpiSurf. The top row highlights the principal stages of the data integration process and the final result, as observed from the interfaces (last two columns). The data sources are listed vertically on the left side.

Figure 2 provides an overview of the steps involved to complete the integration process, including the mapping of metadata (column Content construction) and transformation/cleaning (Content curation).

### 3.4. Sequence Analysis

In this second stage, the pipeline focuses on filling the information in the *blue* portion of VCM (Figure 1) by analyzing each nucleotide sequence and finding the changes with respect to the reference genome that gives the viral sequence peculiar characteristics. First of all, the pipeline performs a global alignment of the sequence to the reference genome (NCBI accession_id NC_045512.2, for SARS-CoV-2 samples) using a slightly modified version of the Needleman–Wunsch algorithm with affine gap penalty (i.e., gaps with length *n* are penalized as gap_open+(n−1)×gap_extension, with |gap_open|>|gap_extension|). In particular, the version of the algorithm we employed is such that, being |G| the length of the reference genome, |S| the size of the target sequence, the dynamic programming matrix $M\in R^{\left(|G|+1)\times (|S|+1\right)}$ is built in such a way that: (a) ∀i∈{2,3,⋯,|G|},M[i,0]=0 (i.e., continuous deletions at the very beginning of the reference genome are not penalized), (b) ∀i∈{2,3,⋯,|S|},M[0,1]=0 (i.e., continuous insertion before the beginning of the reference genome are not penalized), (c) ∀i∈{2,3,⋯,|G|−1}, moving from M[i,|S|] to M[i+1,|S|] has a cost of zero (i.e., continuous deletions at the end of the reference are not penalized) and finally (d) ∀i∈{2,3,⋯,|S|−1}, moving from M[|G|,|i|] to M[|G|,i+1] has a cost of zero (i.e., continuous insertion at the end of the reference are not penalized). Nucleotide changes are extracted from the two aligned sequences and labeled as insertions, deletions, and substitutions; finally, nucleotide changes are processed through snpEff [17] for computing the putative effects over the protein’s stability. This piece of information appears in the VCM as VariantImpact.

Then, to compute the amino acid changes and annotations of an isolate, the pipeline translates the nucleotide sequence corresponding to each of the coding regions into the corresponding amino acid sequences, aligns it with the amino acid sequence of the reference (again using the Needleman–Wunsh algorithm), and, finally, extracts the changes with respect to reference genome (insertions/deletions/substitutions).

#### Epitopes’ Integration

From the IEDB Database Export site (https://www.iedb.org/database_export_v3.php (accessed on 30 January 2022)) at the section “CSV Metric Export”, the pipeline downloads CSV exports regarding epitopes relevant for antibodies of class B, T, and MHC ligand. Each file is interpreted as conventional tabular data after extraction, allowing for quick selection of the relevant attributes. Almost all of the attributes in our database are copied *as is* from the source, except for a few characteristics associated with the assay. The attributes assay, assay_type, hla_restriction, and mhc_class, in our database, are obtained as aggregates over the original epitopes of the same assay type; similarly, the response_frequency_pos is computed as an aggregate of the positive tests that yielded the epitope.

The pipeline links each epitope from the Epitope table to a tuple of foreign keys describing the virus, the protein, and the host species. The first two relations are built by mapping the original epitope’s organism name to the organism’s ‘id’ in the Virus table and the original UniProtID to the product in the Annotation table. The third relation connects an epitope to a host of the HostSpecies table. While processing the epitopes, the pipeline also takes care of importing the details of any novel species—those for which we do not have any sequence already—from the NCBI taxonomy database in the table HostSpecies.

In general, there is a simple one-to-one relationship between the host species described in the IEDB records (carefully classified by entities of the ontology ONTIE, https://ontology.iedb.org/ (accessed on 30 January 2022) and those present in the NCBI Taxonomy DB. Sometimes, epitopes refer instead to taxa not included in NCBI; in such cases, it is associated to the host_id of the closest common ancestor, obtained by recursively traversing the ancestry of an ONTIE taxon until corresponding entity in the NCBI Taxonomy DB is found. Nevertheless, the original host information is always conserved inside the attributes source_host_iri and source_host_name of the Epitope table.

Finally, all epitope sequences, whether linear or fragmented, are inserted inside the EpitopeFragment table and joined to the Epitope table through the key epitope_id. As a result, a simple join between the two tables allows us to obtain all the epitope sequences along with their properties.

### 3.5. Database Finalization

Some steps of the pipeline require a full scan of the local repository. During these operations, the integration of new sequences is paused until the database is finalized. The finalization process involves many tasks: (i) annotate sequences imported from NCBI and NMDC with the lineage information computed by Pangolin [18] (sequences imported from other sources have it pre-computed); (ii) identify the duplicate sequences, i.e., the sequences that have been submitted to more than one database; (iii) import epitopes from IEDB and fill the content of the tables Epitope, EpitopeFragment, Epitope<Virus><Product> necessary for EpiSurf; (iv) update the materialized view NucleotideVariantAnnotated which lists the mutated genes of each sequence; (v) clone the databases to create a stable production version of the repository, ready for the web interfaces ViruSurf, EpiSurf, and VirusViz. In the rest of this section, we provide further details about some of the aforementioned operations.

#### 3.5.1. Lineage Annotation

Unlike the sequences coming from GISAID, NMDC, or COG-UK, the ones of NCBI do not come with the assigned lineage. To increase the homogeneity between the sources, we compute such information with the help of the Pangolin, the same tool used by the other sources to assign the lineage of each sequence. Pangolin command-line tool that accepts a Multi-FASTA file as input, including all nucleotide sequences of the isolates to annotate. We prepare such files via a proper query to our database. After running Pangolin on the Multi-FASTA file, we obtain a CSV file that associates each of the sequences’ accession_id with the corresponding lineage. We then parse the output of Pangolin and load its useful content into our database.

#### 3.5.2. Duplicate Sequences

As some sequencing laboratories submit the same sequence to multiple database institutions, after the sequence import phase, we scan our integrated collection to detect isolates that have been imported twice. The sequences are compared using the attributes strain_name, length and optionally also the geolocation. While for geolocation attributes and length we perform simple equality tests, the matching of the strain_name record is done via a string inclusion test, in conjunction with custom rules derived from the knowledge of the sources to reduce false positives. The result of the comparison is stored internally as a set of pairs of accession_id in the dedicated table Overlaps. The Overlaps table allows to significantly reduce the workload of finding the duplicates, since only the unlisted sequences should be checked again in a subsequent scan.

An additional advantage of this process is the possibility of increasing the completeness of the data by merging the information available in multiple sources. This feature is particularly important for COG-UK isolates because they often lack the content of the attributes submission_date, sequencing_lab, originating_lab, isolation_source, and is_complete. Thanks to the overlaps detection, when a pair of overlapping sequences is found, the metadata values from other sources are used to enrich the corresponding sequence in COG-UK.

#### 3.5.3. Measure of Eptiopes’ Stability

Epitopes are the main focus of the web application EpiSurf. The application, however, is not just another interface over IEDB epitopes, but provides additional statistics over the sequences’ mutations that could be dangerous for the epitope’s recognition and the diagnosis of the infection. The calculation of these is a computationally expensive operation that cannot be addressed at runtime because of the high number of sequences, mutations, and epitopes regarding SARS-CoV2. Instead, the pipeline calculates them offline and stores the result into several tables, one for every combination of virus and protein, comprehensively indicated as Epitope<Virus><Product> in the schema of Figure 1. Each row of these tables links an epitope region—or fragment, if the epitope is non-linear—with one amino acid variation of the previously imported sequences (fully described through its *id*, reference and alternative alleles, position, length, and type of change), the name of the mutated protein and the foreign keys pointing to the sequence owning the change, the species of the viral sequence, and the host organisms.

#### 3.5.4. Gene Annotations

Genes from the Annotation table and nucleotide changes from table NucleotideMuation are pre-joined into a materialized view that supports the fast addition of constraints on the nucleotide sequence from the interface of ViruSurf and EpiSurf. For each sequence, this data structure (called NucleotideVariantAnnotated) stores the gene’s name and the nucleotide changes inside the gene.

#### 3.5.5. Query Optimization

As shown in the Viral Conceptual Model, which is similar to Genomic Conceptual Model [19], all the interfaces have been implemented with a query optimization similarly to GenoSurf [20]. When a query is composed of the interface of ViruSurf or EpiSurf, the query is translated down to a SQL query in the back-end. At every interaction of the user with the drop-down filters present in the interface, we query the smallest subset of tables that are necessary to update the numeric values located inside the drop-down menus, that indicates the number of sequences that are being included by each selected category. Additionally, the result table of each interface is obtained through a join between the Sequence table and the tables whose attributes are being filtered by the specific user-query; for example, if the user did not specify any mutation, the SQL query does not include any of the analytical perspective tables.

### 3.6. Incremental Update

During the last year, sequencing laboratories have become more efficient in collecting and sharing isolates. Indeed, the amount of isolates submitted to database institutions has been growing at an incredibly high rate. This puts us in front of the need to integrate huge data volumes and to do it rapidly. We cope with such requirements by applying both software and hardware strategies that improve the performance of the pipeline. From the hardware point of view, we moved from a single-server architecture serving concurrently the ViruSurf’s interface and running the pipeline to a two-node architecture that can distribute the workload more efficiently. Now the pipeline can run full time on a separate machine with dedicated resources, without affecting the performance of any other service. As for the software, the pipeline for COGUK’s and NCBI’s data (these are the more computationally demanding sources because they are frequently updated and require the calculation of all the parts of the analytical dimension of the VCM) uses a multi-process implementation that assigns the burden of aligning, annotating, and analyzing the sequences to several worker threads, while the main thread takes care of the download, metadata-transformation, and database-loading parts. Additionally, having to process hundreds of thousands of sequences from NCBI, COGUK, and GISAID, it would require too much time to integrate them all at once before every new release of ViruSurf. Instead, we opted for a continuous integration paradigm, through which we progressively build a new release, starting from the most recent one. Indeed, the pipeline keeps updating the very same databases over time and interrupts only for the finalization steps, with which a copy of the working databases is created, optimized as described in the previous sections, and finally transferred to the server responsible for the web interfaces. After the transfer, the pipeline resumes the updates from the point it was interrupted. Updates for the local repository are determined by comparing the accession_id of the sequences present in the database with those available from the sources, obtaining three sets: (a) the sequences that are present and identical in both places, (b) those that are missing in the local database and must be added, and (c) those that are missing in the sources and must be removed. When updating the sources GISAID and COG-UK, comparing only the accession_id is insufficient because the isolates’ metadata change frequently (e.g., a new, more specific clade or lineage appears) and sometimes, even the nucleotide sequences and their amino acid changes can show small differences when observed at different times. Consequently, we also compare several other attributes for these sources, including length, GC%, N%, lineage, strain, amino acid changes (only for GISAID), host attributes (country, region, isolation_source), sequencing project attributes (submission_date and sequencing_lab), and the nucleotide sequence—only if we did not observe any difference in the previous attributes of the Sequence. The result of the comparison generates two additional sets of sequences that are present both locally and in the source of origin but are not identical: (d) the ones whose changes in the source do not affect the Sequence entity, and (e) those that instead affect. Such a difference is relevant because the pipeline can quickly update the isolates of the group (d) without the need for deleting and then re-importing all the parts of the VCM schema concerning those sequences. Differently, group (e) isolates require the ex novo analysis of nucleotide changes, annotations, effects, amino acid changes, overlaps, and possibly other attributes. It is more convenient to remove and re-import them from scratch than updating the database (the computational cost is equivalent). Once the above sets are defined, the pipeline starts an update cycle, which begins by removing the sequences of the sets (c) and (e) proceeds by updating the isolates in the set (d), and finally integrates the sequences from sets (b) and (e). If the cardinality of the set (b) + (e) is greater than few tens of thousands, the pipeline limits the number of sequences imported in one time to reduce the probability of a future update occurring in the sequences that have been just integrated; i.e., it is more convenient to restart the pipeline frequently and always compare the local repository with the latest updates than spending time on synchronizing with a snapshot of a source that may become quickly outdated. The sequences exceeding the threshold set for the cardinality of (b) + (e) will be imported in the next update cycle and, after a new evaluation of the sets, defining the updated/novel/outdated isolates. Update cycles are usually launched daily, except during the finalization of the databases.

### 3.7. Full Automation of Pipeline Operations

The process of updating and periodically deploying a new data release requires pausing the integration process, performing multiple finalization steps—we discussed them in the previous sections—transferring the repository to a different server and finally resuming the pipeline. It involves the coordination of numerous steps and technologies. Most of the operations concerning the integration of sequences and epitopes and the management of duplicates are accomplished through our pipeline written in Python. Others need to resort to external tools, such as SnpEff and Pangolin. Some more are completely dependent on the database server and are executed through SQL scripts. We developed a set of Bash scripts to manage all these tasks with the goal of reducing downtime and manual intervention to the bare minimum. Such scripts are able to launch the integration pipeline and alternate the sources in use, updating sequences and epitopes automatically. Periodically, a function scheduled in the system temporarily pauses the integration process to finalize the repository and transfers it to the server responsible for the interfaces of ViruSurf, EpiSurf, and VirusViz. In conclusion, we are able to periodically generate a new database release without human intervention in a completely autonomous way. This feature can bring a significant reduction of the maintenance time and an increase in the throughput, calculated as the number of sequences integrated over time.

## 4. Results

We report some measurements useful for evaluating the methods previously described.

### 4.1. Query Performance Metrics

In this section, we test database performances, in particular showing the efficiency gain obtained by pre-computing the annotations relative to nucleotide variants and epitopes’ stability measures, as described in Section 3.5.3 and Section 3.5.4. Database performances have been measured by executing a sample set of queries on a database including only GenBank’s, NMDC, and COG-UK’s data and considering only the SARS-CoV-2 sequences collected from human hosts, which is the largest set of sequences. The cardinalities of the VCM tables present in the test database are reported in Table 1.

The first set of queries (named as *Qg1–3*) are concerned with the annotation of nucleotide variants on the viral genome. Table 2 describes in natural language three queries that require the alignment of nucleotide variants and genes. Those queries are optimized by the relation NucleotideVariantAnnotated; such a materialized view, indeed, stores the result of a join between the content of the table NucleotideVariant and the attribute gene_name from the Annotation table. The timings of the queries *Qg1–3* are reported in Table 3.

The second set of queries is named *Qe1–5* and is focused on the relation between the tables Epitope, EpitopeFragment AminoAcidVariant, HostSpecie, Virus, and the attributes product and sequence_id from tables Annotation and Sequence, respectively. The queries, described in natural language in Table 4, have been executed first without any optimization, by joining only the tables—corresponding to the single entities of the VCM schema—necessary for the goal and, then, by using the optimized tables of epitopes’ stability—their timings and a rough estimate of the sequences and epitopes considered to answer each query are listed in Table 5.

**Figure 3:**
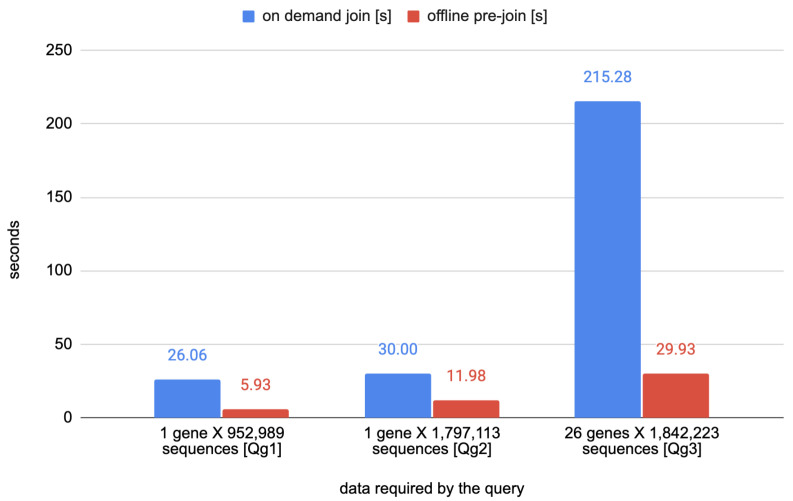
Comparison time required for executing the queries *Qg1–3*.

**Figure 4:**
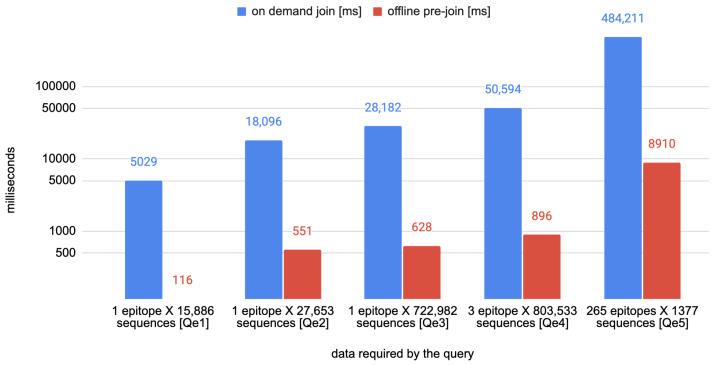
Comparison of the time needed for executing the queries *Qe1–5* with and without the use of the optimized epitopes’ stability tables.

The plots of Figure 3 and Figure 4 visually show the advantage of the mentioned optimizations.

### 4.2. Pipeline Performance Metrics

**Figure 5:**
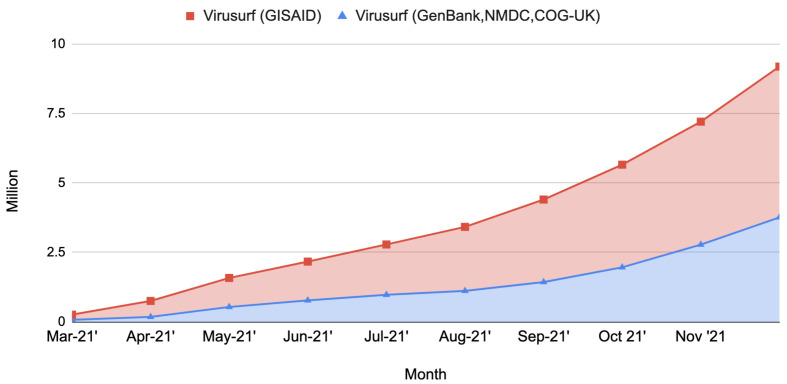
Stacked area chart showing the number of sequences imported in ViruSurf at the conclusion of each month. An increase is clearly noticeable in the performance obtained toward the end of the year 2021, corresponding to the latest improvements on both the hardware and software sides. Records about the number of sequences imported are available only from March 2021.

The data pipeline has been working since late August 2020. Successive refinements brought to increased efficiency obtained by introducing software and hardware improvements. Especially on the hardware side, after August 2021 we moved the integration pipeline to a dedicated machine hosted by Amazon Web Services (AWS). This configuration allowed us to increase the available parallelism and the final throughput. The current machine is a memory-optimized instance called “r5.metal”, embedding 96 vCPUs and 768 GB of RAM. On the software side, we implemented several scripts that manage the start, stop, and restart of the pipeline stages automatically, reducing manual operations and idle times to the bare minimum. In Figure 5 we give an overview of the performance of the sequence integration pipeline in terms of number of sequences imported per month.

## 5. Discussion

In 2020, at the beginning of the COVID-19 pandemic, we started the creation of a bioinformatics pipeline to process and integrate sequences of SARS-CoV-2 submitted to the most relevant genomic database institutions. We adopted a relational model, the Virus Conceptual Model, as a comprehensive and extensible representation of isolates. Such a model allowed seamless integration, and the epitope annotations provided by IEDB. During the year 2021, the number of SARS-CoV-2 sequences greatly increased. Considering only the NCBI data source, at the end of 2020 there were only 47,193 SARS-CoV-2 sequences available, while, at the end of 2021, they became 3,018,196. Over time, we introduced dedicated database structures to allow fast queries even on a repository counting terabytes of curated data (offline pre-calculation of epitope mutations and nucleotide variant gene annotations). We moved from periodically creating new—updated—databases to the release of snapshots of a single continuously updated repository. There were also changes on the hardware side, moving the computational cost of running the pipeline to a dedicated server to avoid excessive resource consumption on the machine supporting the interfaces of ViruSurf, EpiSurf, VirusViz, and others services. Additionally, we automated all the operations of snapshots management, including the creation of the repository images, their transfer, the update, and the finalization of the databases, drastically reducing the idle times and increasing the efficiency. All these improvements contribute to address the concerns of scalability and performance raised by the need of integrating huge amounts of data.

Since the pandemic’s beginning, we periodically produced an updated and integrated repository that comprised almost all the sequences collected worldwide and regularly updated epitope definitions. Currently, the pipeline generates a repository of integrated sequences distributed in two databases: one comprising more than 5.4 million SARS-CoV-2 sequences imported from GISAID, and another database for NMDC, COG-UK, GenBank, and RefSeq with 3.7 million COVID-19 sequences, plus 35 thousand sequences of other viral species. Both databases contribute to the creation of one of the largest integrated repositories of viral sequences and support the following applications.

### 5.1. ViruSurf

The collection of integrated sequences powers the web application ViruSurf with regularly updated isolates of SARS-CoV-2, SARS-CoV, MERS, Dengue, and Ebola collected from humans and several animal species. Through the interface of ViruSurf, it is possible to build complex queries easily, combining the filters available on the biological, organizational, experimental, and analytical dimensions of the VCM. Moreover, it is possible to export query and query results in order to integrate ViruSurf within external tools or to monitor a population of interest by repeating the same query over time. ViruSurf and a GISAID-specific version of ViruSurf are available at http://gmql.eu/virusurf/ and http://gmql.eu/virusurf_gisaid/ (both accessed on 30 January 2022), respectively.

### 5.2. EpiSurf

ViruSurf’s extension, EpiSurf, leverages the integration of IEDB’s epitopes to let anyone study the conservancy of these peptidic regions in a user-defined population through simple-to-use filters on the properties of the isolates and on the epitopes. As in ViruSurf, the user can combine filters on the metadata and the sequence’s characteristics to select the population of interest. Upon selecting the protein and the epitopes to study, a table summarizes, for each epitope, statistics about the overlapping amino acid changes and about the isolates that carry such alterations. The user can choose to analyze a subset of the already integrated IEDB epitopes or provide their regions of interest, expressed as ranges over the translated reference sequence. It is possible to search sequences of NCBI, COG-UK, and NMDC through EpiSurf at http://gmql.eu/episurf/, and GISAID sequences on a dedicated interface available at http://gmql.eu/episurf_gisaid/ (both accessed on 30 January 2022).

### 5.3. Visurviz and VirusLab

VirusViz [21] is a visualization and analysis tool available at http://gmql.eu/virusviz/ (accessed on 30 January 2022) integrated with ViruSurf and EpiSurf. Through them, the user can select a population of interest and explore the extracted sequences in VirusViz, visualize their amino acid or nucleotide changes aligned on the reference genome, see how they are distributed, and group the sequences according to any metadata attribute. VirusViz is designed to explore the isolates in our repository, but can also import external datasets described as a Multi-FASTA file for the nucleotide sequences, plus an additional CSV file for metadata; in this case, a simplified version of the pipeline hereby presented (including sequence analysis and basic metadata identification) processes the external sequences and produces gene annotations, protein annotations, amino acid changes, nucleotide variations, and prediction of functional effects. A copy of our pipeline is also powering VirusLab [3], a private version of VirusViz, designed to be used in environments with strict privacy concerns, such as hospitals, for analyzing the viral sequences sampled from local patients.

In conclusion, this work made it possible to create valuable data exploration and analysis tools like ViruSurf, EpiSurf, VirusViz, and VirusLab, and maintains a genomic data repository of paramount importance for anyone interested in studying SARS-CoV-2 or other viral species harmful to humans.

## Figures and Tables

**Table 1:** Cardinalities of the tables (without the optimization data structures) included in the test database.

Entity	Cardinality	
virus soecies	13	
host samples	118,217	
hosts	80	
sequencing projects	5270	
experiment types	12,996	
	SARS-CoV-2	all other viruses
sequences	1,808,481	35,340
amino acid variants	77,005,463	61,501,438
annotations	70,370,040	268,326
variant effect predictions (variant impacts)	343,024,713	13,308,703
nucleotide variants	60,281,229	7,882,613
epitopes	6557	11,558
epitope fragments	8063	12,336

**Table 2:** Examples of queries optimized by the materialized view NucleotideVariantAnnotated; the view’s definition implements the join operation between the table NucleotideVariant, and the attribute gene_name from table Annotation offline.

Query	Task
Qg1	Sort sequences’ lineages by the number of mutation falling in gene ‘M’
Qg2	Sort the nucleotide variants falling in gene ‘S’ by number of occurrences
Qg3	Find the most frequently mutated genes

**Table 3:** Execution times and rough estimate of the resources involved for running the queries *Qg1–3* of Table 2.

Query	Data Required by the Query	Execution Time [s]	
		On Demand Join	Offline Pre-Join
Qg1	1 gene × 952,989 sequences	26.06	5.93
Qg2	1 gene × 1,797,113 sequences	30.00	11.98
Qg3	26 genes × 1,842,223 sequences	215.28	29.93

**Table 4:** The five test queries used for measuring the performance increase given by the offline pre-computation of the sequences mutating epitope regions. All the queries are relative to SARS-CoV-2 viral samples collected from humans.

Query	Task
Qe1	Count sequences harbouring a mutation on the epitope 1075107 (iedb_epitope_id)
Qe2	Select distinct lineage, country, collection_date of viral sequences with mutations in regions of the protein NS7a corresponding to epitopes having response frequency >0.555
Qe3	Rank amino acid changes by mutation rate of a specific epitope (iedb_epitope_id 1075107)
Qe4	Rank amino acid changes by mutation rate of the set of epitopes on protein NS8 having response frequency > 0.74
Qe5	Select distinct epitopes in protein M mutated in human hosts by sequences of lineage B.1.177

**Table 5:** Performance measurements of the queries *Qe1–5*. The measurement in the column “On-demand join” are obtained by running the queries without using the pre-computed tables of mutation and epitopes. The following columns report the timings observed when executing the same queries using the pre-computed tables.

Query	Data Required by the Query	Execution Time [ms]	
		On Demand Join	Offline Pre-Join
Qe1	1 epitope × 15,886 sequences	5028.68	115.78
Qe2	1 epitope × 27,653 sequences	18,096.08	550.85
Qe3	1 epitope × 722,982 sequences	28,182.36	628.37
Qe4	3 epitope × 803,533 sequences	50,594.49	895.99
Qe5	265 epitopes × 1377 sequences	484,210.50	8910.01

## Data Availability

The pipeline described in this document periodically produces an integrated collection of viral sequences that is accessible at the websites http://gmql.eu/virusurf, http://gmql.eu/virusurf_gisaid. The collection of sequences and epitopes is instead available at http://gmql.eu/episurf, and http://gmql.eu/episurf_gisaid. The software corresponding to the integration pipeline is hosted at the GitHub repository https://github.com/DEIB-GECO/virusurf_downloader. All the links have been accessed on 30 January 2022.
